# A comparative study of multiple neural network for detection of COVID-19 on chest X-ray

**DOI:** 10.1186/s13634-021-00755-1

**Published:** 2021-07-27

**Authors:** Anis Shazia, Tan Zi Xuan, Joon Huang Chuah, Juliana Usman, Pengjiang Qian, Khin Wee Lai

**Affiliations:** 1 Department of Biomedical Engineering, Faculty of Engineering, Universiti Malaya, 50603 Kuala Lumpur, Malaysia; 2 Department of Electrical Engineering, Faculty of Engineering, Universiti Malaya, 50603 Kuala Lumpur, Malaysia; 3 School of Artificial Intelligence and Computer Science, Jiangnan University, 1800 Lihu Avenue, Jiangsu 214122 Wuxi, People’s Republic of China

**Keywords:** Artificial neural networks, Deep learning, Transfer learning, Multi-task learning, COVID-19, Classification, DenseNet121

## Abstract

Coronavirus disease of 2019 or COVID-19 is a rapidly spreading viral infection that has affected millions all over the world. With its rapid spread and increasing numbers, it is becoming overwhelming for the healthcare workers to rapidly diagnose the condition and contain it from spreading. Hence it has become a necessity to automate the diagnostic procedure. This will improve the work efficiency as well as keep the healthcare workers safe from getting exposed to the virus. Medical image analysis is one of the rising research areas that can tackle this issue with higher accuracy. This paper conducts a comparative study of the use of the recent deep learning models (VGG16, VGG19, DenseNet121, Inception-ResNet-V2, InceptionV3, Resnet50, and Xception) to deal with the detection and classification of coronavirus pneumonia from pneumonia cases. This study uses 7165 chest X-ray images of COVID-19 (1536) and pneumonia (5629) patients. Confusion metrics and performance metrics were used to analyze each model. Results show DenseNet121 (99.48% of accuracy) showed better performance when compared with the other models in this study.

## Introduction

The novel coronavirus of 2019, or simply known as the COVID-19, affects the respiratory tracts and the lungs leading to severe cases of pneumonia. The usual symptoms include fever, dry hack cough, body ache, and loss of taste or smell. In extreme cases, the patient may experience shortness of breath and multiple organ failure and may lead to fatality (https://www.worldometers.info/coronavirus/). While the world pharmaceutical companies are trying to develop vaccination to prevent the spread of this pandemic, the current medical practice to control the spread of COVID-19 is focused on early detection and isolation of the patient. The current gold standard for COVID-19 detection is the real-time reverse transcription-polymerase chain reaction (RT-PCR), where the short sequences of DNA or RNA are reproduced or amplified and analyzed [1]. Fang et al. [2] reported that the RT-PCR testing has a low sensitivity of 71% while Williams et al. [3] reported that the sensitivity of a single RT-PCR test in hospitalized patients is 82.2%.

Perceiving the limitations of RT-PCR, there is a need for cross-verification examination by using radiological images. Chest radiography, particularly chest X-ray, is one of the most frequently performed diagnostic examinations even in underdeveloped areas. Radiographic scanning was proposed to detect pathological effects of COVID-19 by examining chest radiological images of lungs of patients [4]. Several studies have shown that changes in chest radiography images such as X-ray and CT scan were noticed even before the appearance of clinical features of COVID-19 [5]. Interpretation of chest X-ray (CXR) and CT scans have widely been done by radiologists to find some visual indicators for COVID-19 infection as an alternative method for rapid screening of infected patients. For early-stage COVID-19 on CXR, peripheral ground-glass opacities are observed which progresses to consolidations at later stages [6, 7]. Since the studies have shown that the abnormalities caused by COVID-19 are visible in chest X-rays, these abnormalities, especially the opacities, are further used to detect COVID-19.

Radiological COVID-19 detection also experiences challenges due to its similar nature and appearance with viral pneumonia radiographs. It requires medical experts to identify the specific radiographic markers to distinguish between the two conditions. With an enormous number of COVID-19 cases suspected daily, it is difficult to assign enough time and resources to individual radiographs. This discrepancy between the available experts and the need of the human expertise has promoted automation and machine learning to fill this much-needed gap [8]. Over the last year, scientists and researchers are unitedly working to automate the detection methods and provide intelligent machines that can easily distinguish infectious COVID-19 cases from other similar appearing cases. This study is conducted to explore these state-of-the-art techniques that have shown promising result and compare it with the same parameters and datasets to identify the best DL model for COVID-19 detection.

## Related work

Jain et al. [9] implemented ResNet-101 in the classification of COVID-19 and viral pneumonia, achieving an accuracy of 97.78%. Che Azemin et al. [10] used pretrained ResNet-101 to detect COVID-19 in CXR with an accuracy of 71.9% as their training dataset was based on airspace opacity instead of confirmed COVID-19 cases. Ismael et al. [11] also used ResNet-50 architecture but only for feature extraction. The extracted features were classified using an SVM classifier with the Linear kernel function and produced high accuracy of 94.7%. Makris et al. [12] fine-tuned several CNN models and compared their performances in classifying COVID-19, pneumonia, and normal images. VGG16 turned out to have the best performance with an overall accuracy of 95.88% in their study. Abbas et al. [13] proposed a new method to classify COVID-19, SARS, and normal CXR which is called DeTraC (stands for Decompose, Transfer, and Compose). It is done by adding a class decomposition layer to the pretrained models that can partition each image class into sub-classes, but assemble back during prediction. By using VGG19 with the DeTraC approach, the model has achieved a classification accuracy of 93.1%. Asif et al. [7] trained InceptionV3 using transfer learning techniques to distinguish COVID-19 from viral pneumonia and normal CXR and obtained an accuracy of 98%. Inspired by DarkNet architecture, Ozturk et al. [14] developed a deep learning network named DarkCovidNet for automated COVID-19 diagnosis. The model achieved an accuracy of 98.08% for binary (COVID-19 and normal) and 87.02% for multiclass (COVID-19, pneumonia, and normal) classification. Shelke et al. [15] worked in the segregation of COVID-19 and normal pneumonia using DenseNet-161 and achieved an accuracy of 98.9%. Minaee et al. [16] fine-tuned 4 pretrained networks (ResNet18, ResNey50, SqueezeNet, and DenseNet-121) and compared their performance. Different cut-off thresholds for probability score were experimented in this study. SqueezNet turned out to be the best model with a sensitivity of 98% and a specificity of 92.9%. Das et al. [17] have developed a new model with a weighted average ensembling method; the model comprises of three pre-trained CNN models—DenseNet201, Resnet50V2, and InceptionV3. This approach has achieved an accuracy of 95.7% and a sensitivity of 98% in the classification of positive and negative COVID-19 cases. Ridhi et al. [18] proposed a new method to classify COVID-19, pneumonia, and normal CXR by using stacked of DenseNet and GoogleNet as feature extractor, and then the features were classified by the ensemble of XGB, RF, and SVM classifiers. The classification accuracy obtained in this study is 91.7%. Gupta et al. [19] proposed an integrated stacked deep convolution network called InstaCovNet-19 which makes use of InceptionV3, NASNet, Xception, MobileNetV2, and ResNet101. The proposed model achieved an accuracy of 99.53% in binary (COVID-19 vs non-COVID-19) classification and an accuracy of 99.08% in 3-class (COVID-19, pneumonia, normal) classification. A 22-layer CNN architecture was proposed by Hussain et al. [20] which achieved a classification accuracy of 99.1%, 94.2%, and 91.2% for binary, 3-class, and 4-class classification, respectively. Canayaz et al. [21] developed a model called MH-COVIDNet that used VGG19 as a feature extractor and BPSO meta-heuristic algorithm (MH algorithm) for feature selection. This approach obtained a classification accuracy of 99.38%. Khuzani et al. [22] performed feature extraction using different techniques such as Texture, FFT, Wavelet, GLCM, and GLDM. In the study, a multilayer network was created with 2 hidden layers of 128 and 16 neurons and a final classifier. The 3-class classification (COVID-19, pneumonia, and normal) has achieved an accuracy of 94%.

From the above researches, it is observed that identification of the novel coronavirus on radiological images using deep learning techniques has the potential to reduce the pressure on radiologists. However, with various researchers using different deep learning methods, it is unclear which model provides the best result. Therefore, this study compares various deep learning models that have given impressive results in COVID-19 identification. In this study, we have fine-tuned existing models (VGG16, VGG19, DenseNet121, Inception-ResNet-V2, InceptionV3, Resnet50, and Xception) based on our classification requirements. These models have shown remarkable results in pneumonia detection [23–25] and have also been showing promising results with COVID-19 [11, 26, 27] classification. Hence, in this study, we have compared them based on the same data and variables to determine the best model to distinguish COVID-19 X-ray from pneumonia. The models have been trained and tested on COVID-19 and pneumonia CXR images from multiple datasets to avoid any biases. The models are then compared based on their performance metrics and computational time taken. The results are carefully analyzed, and the best model is chosen for this binary classification.

## Materials and methods

### Dataset

Due to the limitation of publicly available COVID-19 data, we have complied with multiple databases for this study. All images collected for pneumonia and COVID-19 are from publicly available datasets. Table 1 tabulates the various databases and the number of images adopted from them; similar images were eliminated. A total of 1536 COVID-19 and 5629 pneumonia images were used for training, validation, and testing of the models. The images collected from these databases were of various dimensions, which was resized to 224 × 224 pixels.

**Table 1:** List of databases used in this study

	Dataset	COVID-19	Pneumonia
1	CoronaHack-Chest X-Ray-Dataset [28]	19	4118
2	Covid_Data_GradientCresent [29]	69	158
3	Covid-19 Radiography Database [30]	1143	1345
4	Covid-chestxray-dataset [31]	142	–
5	Figure1-COVID-chestxray-dataset [32]	35	–
6	Actualmed-COVID-chestxray-dataset [33]	58	–
7	COVID-19-x-ray-10000-images [34]	70	–
8	ChestXRay2017 [35]	–	8
	Total	1536	5629

From the total samples of COVID-19, 10% of samples was randomly selected for testing. The remaining sample was split into 80% for training and 20% for validation. Similarly, a balanced dataset was obtained by randomly selecting a similar number of samples for training and validation of pneumonia and splitting them 80% for training and 20% for validation. The remaining samples were used for testing. Table 2 tabulates the total images used in each class used for training, validation, and testing. The training and validation tests were balanced to obtain a better result and to avoid overfitting to the majority class that is the pneumonia cases. A balanced training set has been observed to give the highest accuracy regardless of the instances in the test dataset [36]. Also, the models were exposed to images from various databases to avoid any biases towards a database. Also, the imbalance between the two test sets was done to imitate a real-life environment where a number of cases are not balanced and are not from one particular source.

**Table 2:** Data for training, validation, and testing

	COVID-19	Pneumonia
Training	1101	1104
Validation	276	276
Testing	156	4249

### Transfer learning approach

There are two types of transfer learning in the context of deep learning, which are feature extraction and fine-tuning. In the feature extraction technique, a pretrained model on some standard dataset such as ImageNet is used, but the top layer, which is used for classification purpose, will be removed. Then on top of the pretrained model, it trains a new classifier to perform classification. The pretrained model without the top classifier is treated as an arbitrary feature extractor in order to extract useful features from the new dataset. In the second approach which is fine-tuning, the pretrained model weights are treated as the initial values for the new training, and they are updated and adjusted in the training process. In this case, the weights are fine-tuned from generic feature maps to specific features associated with the new dataset. The goal of fine-tuning is about adapting the generic features to a given task rather than overwriting the generic learning.

For this study, a transfer learning approach was adopted and pre-trained weights from ImageNet were used to compensate for the small training data set. With transfer learning, the models were prevented from overfitting due to the small data set. In this study, we fine-tuned the last layer of seven state-of-the-art deep learning models—VGG16, VGG19, DenseNet12, Inception-ResNet-V2, InceptionV3, ResNet50, and Xception—while using the pre-trained model as a feature extractor. To fine-tune these models for binary classification, the last set of layers which consists of fully-connected layers along with softmax activation function were replaced with a flatten layer, which converts the data from the previous layer to a giant 1-dimensional tensor. A dropout of 0.5 was added for regularization, and lastly, a dense layer was added which applied softmax activation on previous layers and produce two outputs of probability for “COVID-19” and “pneumonia” classes. The next section will briefly discuss the architecture of these models and how they are used for this binary classification.

#### VGG16

**Fig. 1:**
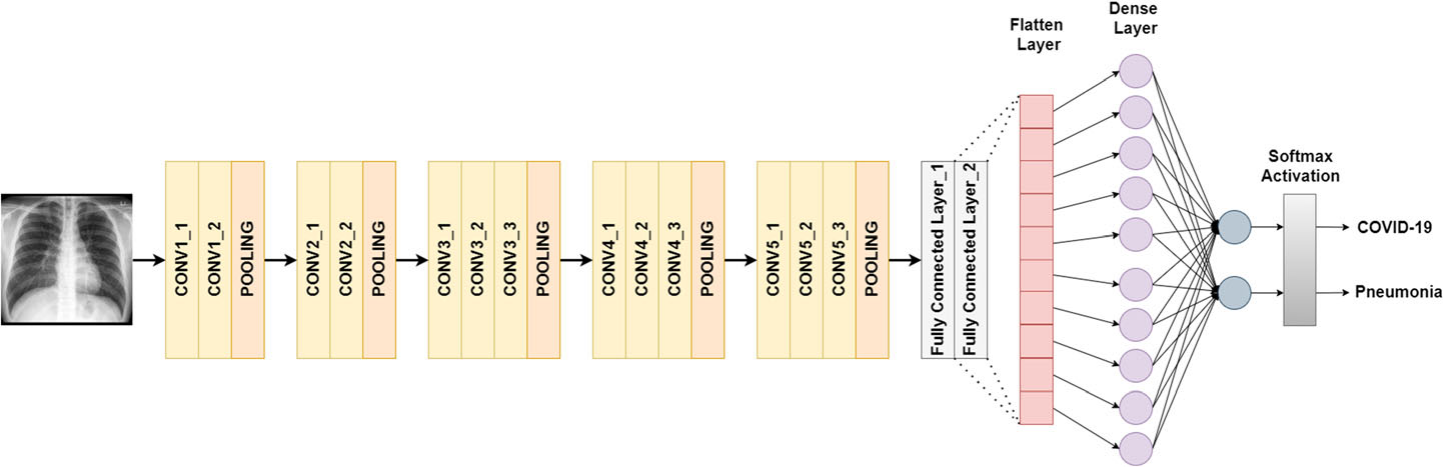
VGG16 architecture designed for binary classification

The input of VGG16 is of fixed size 224 × 224 RGB image. It consists of 16 layers which include 13 convolutional layers and 3 fully connected layers, including max-pooling to reduce the volume size and softmax classifier following the last fully connected layer. For this study, the last fully connected layer along with softmax activation is replaced with our designed classifier as shown in Fig. 1.

#### VGG19

**Fig. 2:**
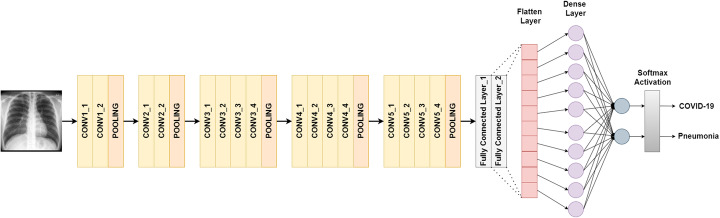
VGG19 architecture designed for binary classification

The input of VGG19 is of fixed size 224 × 224 RGB image. It consists of 19 layers which include 16 convolutional layers and 3 fully connected layers, including max-pooling to reduce the volume size and softmax classifier following the last fully connected layer. For this study, the last fully connected layer along with softmax activation is replaced with our designed classifier as shown in Fig. 2.

#### DenseNet121

**Fig. 3:**
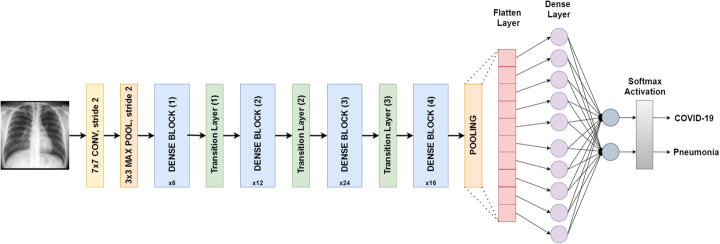
DenseNet121 architecture designed for binary classification

The input of DenseNet121 is of fixed size 224 × 224 RGB image. DenseNet121 consists of 121 layers with parameters of more than 8 million. It is divided into DenseBlocks where the dimensions of the feature maps are the same within the block but the number of filters is different. The layers between the blocks are called transition layers and they apply batch normalization for down-sampling. For this study, the last fully connected layer along with softmax activation is replaced with our designed classifier as shown in Fig. 3.

#### Inception-ResNet-V2

**Fig. 4:**
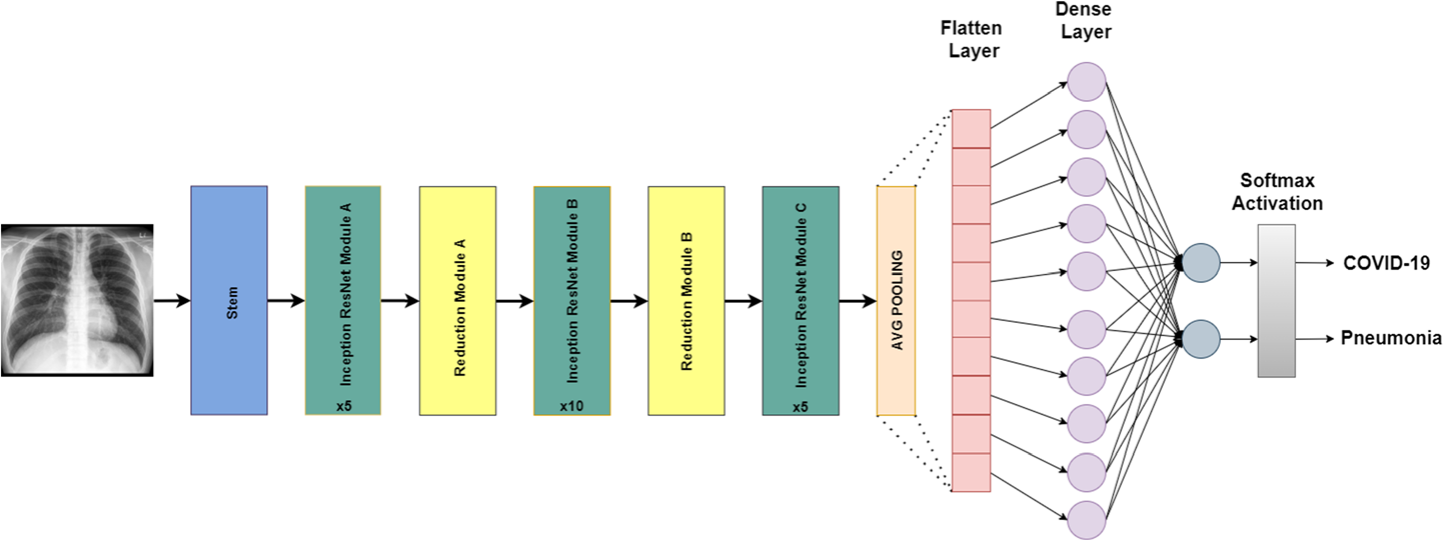
Inception-ResNet-V2 architecture designed for binary classification

The basic building block of Inception-ResNet-V2 is called Residual Inception Block. A 1 × 1 convolution filter expansion layer is used after each block to scale up the filter bank dimensionality before the addition to match the depth of the input. This architecture uses batch normalization only on top of the traditional layers. Inception-ResNet-V2 is 164 layers deep and has an image input size of 299 × 299. The Residual Inception Block incorporates multiple-sized convolutional filters with residual connections. With the use of residual connections, this architecture prevents the problem of degradation due to deep networks and reduces the duration of training. Figure 4 explains our fine-tuned model of Inception-ResNet-V2 for COVID-19 and pneumonia classification.

#### InceptionV3

**Fig. 5:**
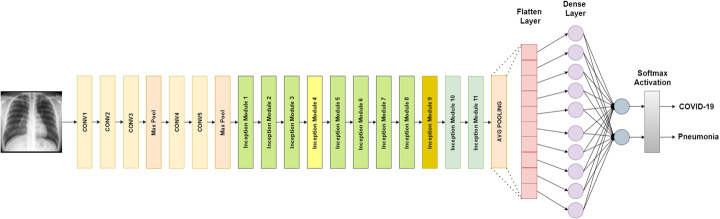
InceptionV3 architecture designed for binary classification

InceptionV3 is made up of 484 layers consisting of 11 inception modules. It has an image input size of 299 × 299. Each module consists of convolution filters, pooling layers, and ReLu activation function. Without downgrading the network efficiency, InceptionV3 reduces the number of parameters by factorizing convolutions. InceptionV3 also proposed novel downsizing to reduce the number of features. Figure 5 explains our fine-tuned model of InceptionV3 for COVID-19 and pneumonia classification.

#### ResNet50

**Fig. 6:**
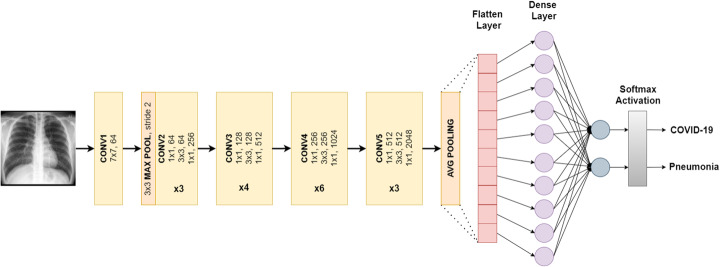
ResNet50 architecture designed for binary classification

ResNet50 is a variant of ResNet or Residual Network. It consists of 48 convolutional layers, 1 MaxPool, and 1 average pool layer. Each convolution block has 3 convolution layers, and there are also 3 convolution layers in each identification block. ResNet-50 has more than 23 million parameters which can be trained. Figure 6 explains our fine-tuned model of ResNet50 for COVID-19 and pneumonia classification.

#### Xception

**Fig. 7:**
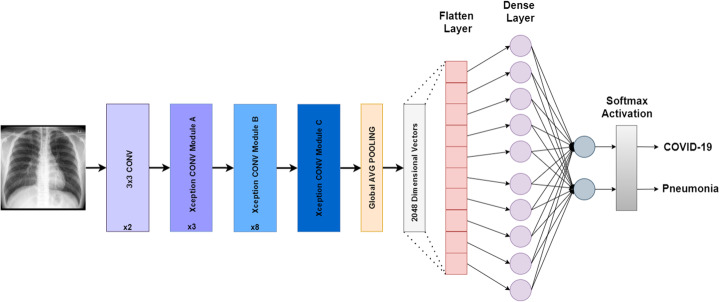
Xception architecture designed for binary classification

Xception was proposed by Chollet in 2016, the creator of the Keras library. It is an adaption of the Inception architectures in which the Inception modules are replaced with depth-wise separable convolutions. Xception outperformed the traditional InceptionV3 with higher Top-1 and Top-5 accuracy on ImageNet dataset. The number of parameters of Xception is roughly the same as InceptionV3 (around 23 million). Figure 7 explains our fine-tuned model of Xception for COVID-19 and pneumonia classification.

#### Model training

For this study, all deep learning models—VGG16, VGG19, DenseNet121, Inception-ResNet-V2, InceptionV3, Resnet50, and Xception—were trained on 12 GB NVIDIA Tesla K80 GPU. All the images of the dataset were resized to 224 × 224 pixels. For the algorithm development and implementation of CNN, the deep learning library – TensorFlow 2.4 with Keras API was used. The model was trained using the categorical cross-entropy loss function to measure the performance of the model from the ground truth probabilities. The categorical cross-entropy loss function is defined as:

1 $$ \mathrm{Categorical}\ \mathrm{cross}-\mathrm{entropy}\ \mathrm{loss}:-{\sum}_{\boldsymbol{c}=\mathbf{1}}^{\boldsymbol{M}}{\boldsymbol{y}}_{\boldsymbol{i},\boldsymbol{c}}\ \mathbf{\log}\left({\boldsymbol{p}}_{\boldsymbol{i},\boldsymbol{c}}\right) $$

where M indicates the class and *y*_*i*, *c*_ and *p*_*i*, *c*_ indicates the ground truth and predicted probabilities for individual images. We then minimized the loss function and improved the efficacy using Adam optimizer with a learning rate of 0.001. We implemented an early stopping technique based on validation performance to overcome the issue of overfit or underfit model. Validation loss was used as a performance measure to terminate the training when no improvement in performance was observed in 20 consecutive epochs.

### Performance metrics

After the models finished training, they were tested on test set to evaluate the model accuracy. The models were tested on 156 COVID-19 images and 4249 pneumonia images. To evaluate the performance of the models, the metrics adopted include overall classification accuracy, recall (also known as sensitivity), precision, and F1-score. The metrics are defined as follow:

2 $$ \mathrm{Accuracy}:\frac{\boldsymbol{TP}+\boldsymbol{TN}}{\boldsymbol{TP}+\boldsymbol{TN}+\boldsymbol{FP}+\boldsymbol{FN}} $$

3 $$ \mathrm{Precision}:\frac{\boldsymbol{TP}}{\boldsymbol{TP}+\boldsymbol{FP}} $$

4 $$ \mathrm{Recall}:\frac{\boldsymbol{TP}}{\boldsymbol{TP}+\boldsymbol{FN}} $$

5 $$ \mathrm{F}1-\mathrm{score}:\frac{\mathbf{2}\times \left(\boldsymbol{Recall}\times \boldsymbol{Precision}\right)}{\left(\boldsymbol{Recall}+\boldsymbol{Precision}\right)} $$

where TP, TN, FP, and FN stand for true positive, true negative, false positive, and false negative. In this study, if the COVID-19 image is correctly classified, it is counted as TP, while if incorrectly classified as pneumonia, it is counted as FN. On the other hand, if a pneumonia image is classified correctly, it is counted as TN and the incorrectly classified as COVID-19 is FP. A confusion matrix was plotted to depict the number of correctly classified images, and a classification report was generated using the scikit-learn metrics function.

## Experimental results and discussions

**Fig. 8:**
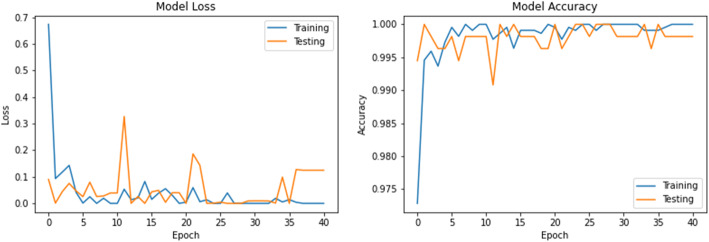
VGG16 accuracy loss graph

**Fig. 9:**
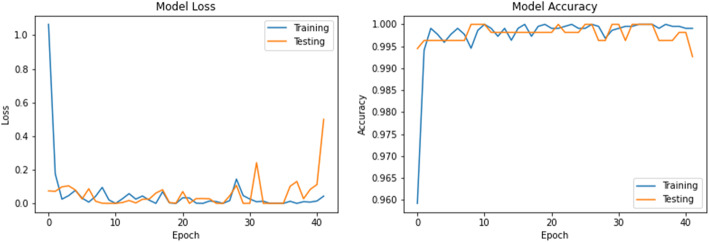
VGG19 accuracy loss graph

**Fig. 10:**
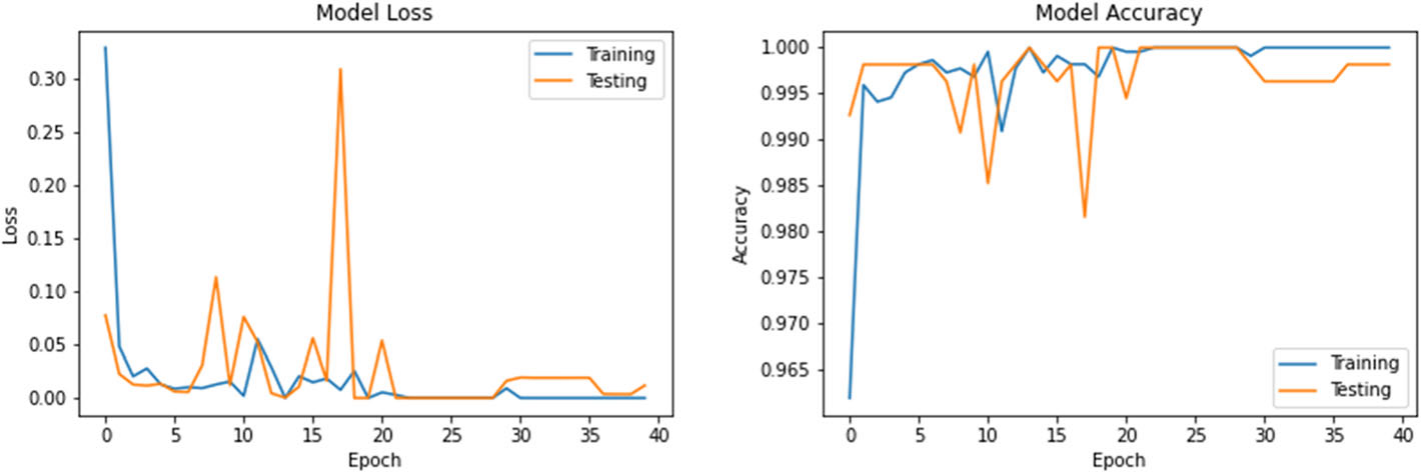
DenseNet121 accuracy loss graph

**Fig. 11:**
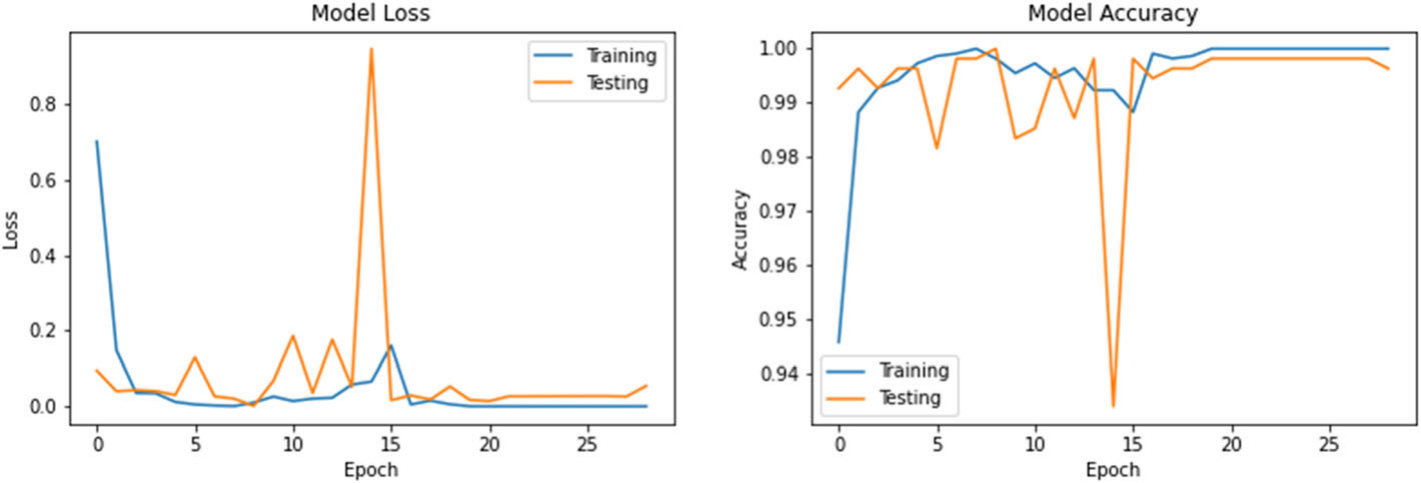
Inception-ResNet-V2 accuracy loss graph

**Fig. 12:**
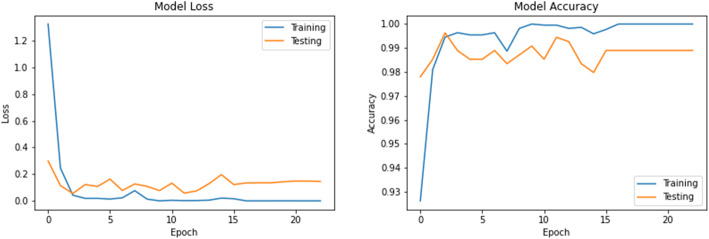
InceptionV3 accuracy loss graph

**Fig. 13:**
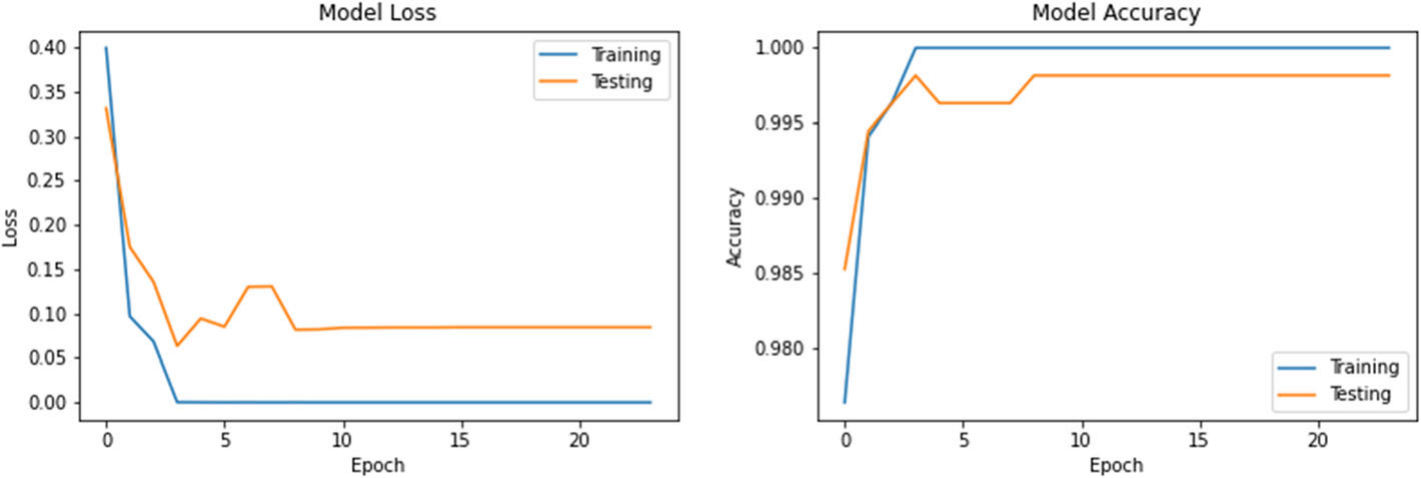
ResNet50 accuracy loss graph

**Fig. 14:**
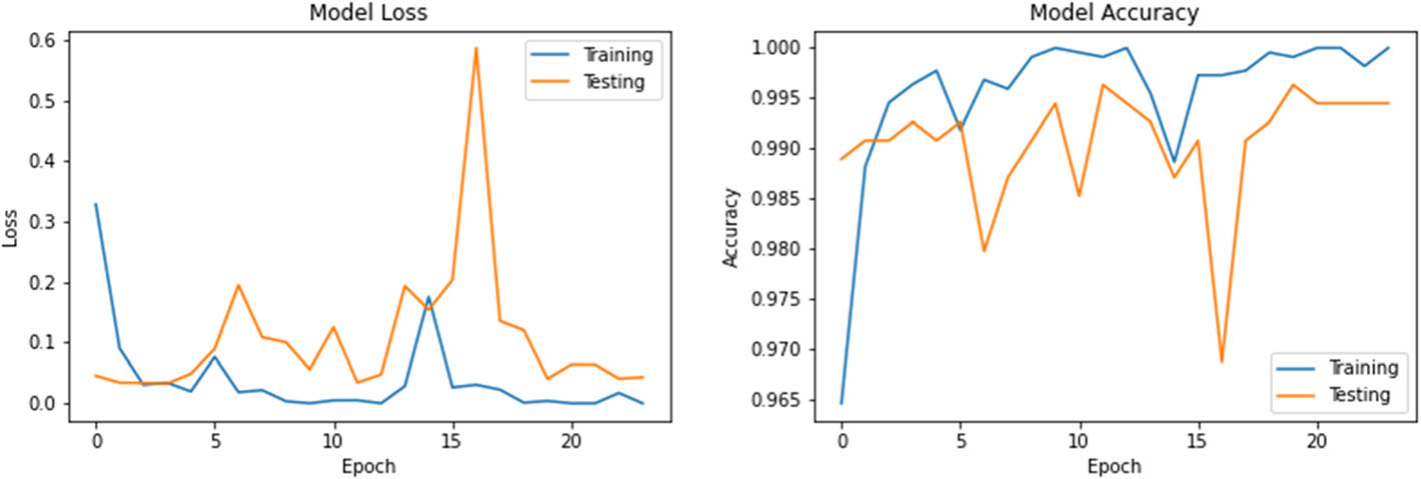
Xception accuracy loss graph

The accuracy and loss values in training and validation process are listed in Table 3 and shown in Figs. 8, 9, 10, 11, 12, 13, and 14 for each fine-tuned model. When comparing the number of epochs taken by each model to reach the minimum validation loss, it is observed that InceptionV3, ResNet50, and Xception reached a minimum loss at just 3, 4, and 4 epochs, respectively. With few epochs, they are able to achieve validation accuracy of 99% and above. This indicates that these models are able to learn the distinctive features between COVID-19 and pneumonia very quickly. However, when loss and accuracy are taken into consideration, it is observed that the training accuracy is highest for DenseNet121 and ResNet50; however, the DenseNet121 has the lowest training loss. For the validation set, VGG16, VGG19, DenseNet121, and Inception-ResNet-V2 have higher accuracy; however, DenseNet121 has the lowest validation loss. Hence, from this data, it can be summarized that the DenseNet121 model exhibits higher training and validation performance among the seven models.

**Table 3:** Accuracy and loss during training and validation

	Best epoch	Training loss	Training accuracy	Validation loss	Validation accuracy
**VGG16**	21	0.0100	0.9990	0.0000e+00	1.0000
**VGG19**	22	0.0122	0.9994	0.0000e+00	1.0000
**DenseNet121**	20	6.1363e−09	1.0000	7.2103e−07	1.0000
**Inception-ResNet-V2**	9	0.0022	0.9993	0.0011	1.0000
**InceptionV3**	3	0.0472	0.9943	0.0554	0.9963
**ResNet50**	4	1.1188e−04	1.0000	0.0633	0.9982
**Xception**	4	0.0430	0.9951	0.0325	0.9926

**Fig. 15:**
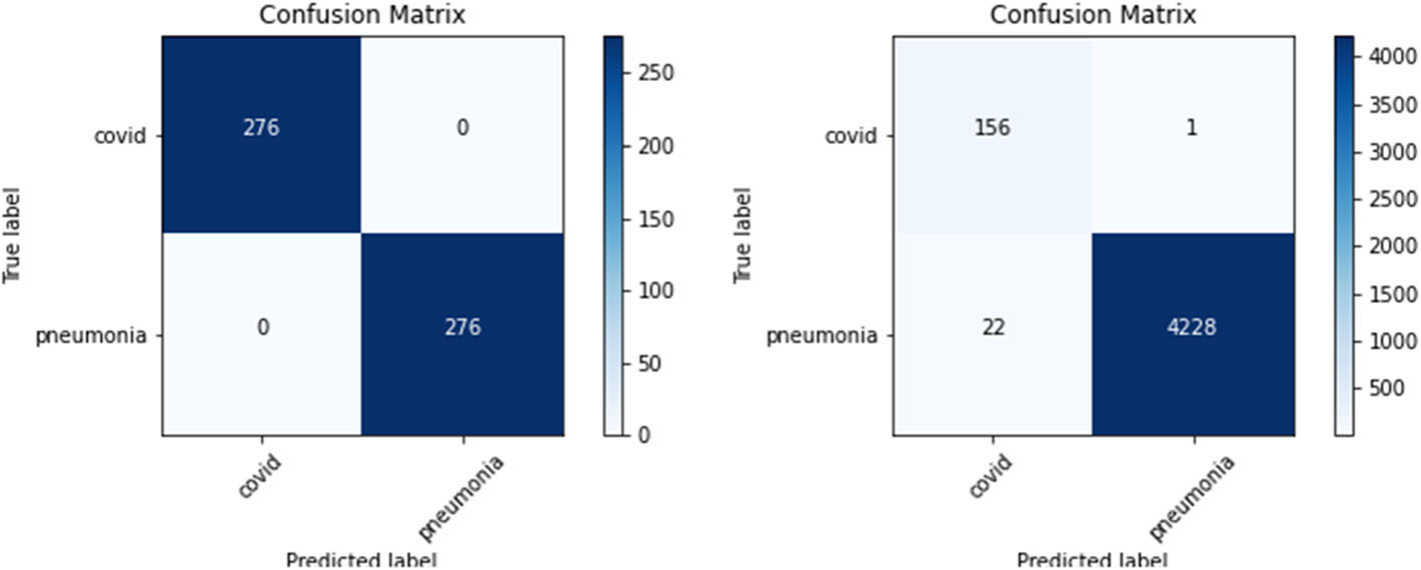
DenseNet121 confusion matrix

The confusion matrix displays numbers of images identified correctly and incorrectly by the model. The confusion matrix was generated for both the validation dataset and the test dataset. The validation dataset comprised of 276 COVID-19 and 276 pneumonia images whereas the test dataset comprised of 157 COVID-19 and 4250 pneumonia images. Table 4 below summarized the confusion matrix for all the seven models. It can be observed that though multiple models performed well during the validation, DenseNet121 has the lowest false positive and false negative, indicating that the DenseNet121 model, as shown in Fig. 15, made the least number of errors while predicting the image was COVID-19 or pneumonia.

**Table 4:** Confusion matrix

Model	Validation				Testing			
	TP	TN	FP	FN	TP	TN	FP	FN
**VGG16**	276	276	0	0	157	4210	40	0
**VGG19**	276	276	0	0	157	4214	36	0
**DenseNet121**	276	276	0	0	156	4228	22	1
**Inception-ResNet-V2**	276	276	0	0	156	4172	78	1
**InceptionV3**	276	274	2	0	155	4206	44	2
**Resnet50**	275	276	0	1	156	4221	29	1
**Xception**	274	274	2	2	155	4179	71	2

This study even compared these pre-trained models based on the accuracy, precision, recall, and F1 score as tabulated in Table 5. It is observed that DenseNet121 gave good classification performance with an accuracy of 99.48%, followed by ResNet50 with 99.32% accuracy. Table 5 also compares the computational times taken by each model for training and testing. It is seen that the InceptionV3 takes the least time (11 min 50 s) for training; however, it is slow during testing (16 min 14 s), whereas DenseNet121 was slower during training (20 min), but it was the fastest during testing (15 min 36 s) with the highest accuracy.

**Table 5:** Performance metrics

Model	Accuracy (%)	Precision (%)	Recall (%)	F1 (%)	Training time (s)	Testing time (s)
**VGG16**	99.09	99.28	99.09	99.14	1353	1012
**VGG19**	99.18	99.34	99.18	99.22	1344	946
**DenseNet121**	99.48	99.54	99.48	99.49	1200	936
**Inception-ResNet-V2**	98.21	98.79	98.21	98.38	899	1083
**InceptionV3**	98.96	99.17	98.96	99.02	710	974
**ResNet50**	99.32	99.42	99.32	99.35	783	1056
**Xception**	98.34	98.83	98.34	98.49	750	865

From the above result, we recommend DenseNet121 (99.48% accuracy, 99.54% precision, 99.48% recall, and 99.49% of F1 score) for classification of COVID-19 from pneumonia cases on chest X-ray, further comparing our fine-tuned DenseNet121 with the studies recently published that also performed binary classification, particularly COVID-19 and pneumonia images. Shelke et al. [15] used a deeper network, DenseNet-161, but the accuracy obtained is lower, which might be due to the lower number of training images. Compared with other works that worked on the binary classification of CXR images, our model has the second highest accuracy (Table 6). The highest binary classification accuracy is obtained by Gupta et al. [19] using their proposed network called InstaCovNet-19.

**Table 6:** Comparison with other related work that performed binary classification on CXR images

Author (year)	CNN architectures	Image classification	Total dataset	Accuracy
Nayak et al. [37]	ResNet-34	COVID-19 and normal	COVID-19: 775 Normal: 775	98.33%
Ismael and Şengür [11]	ResNet50 feature + SVM	COVID-19 and normal	COVID-19: 180 Normal: 200	94.7%
Ozturk et al. [14]	DarkCovidNet	COVID-19 and no-findings	COVID-19: 177 No-findings: 500	98.08%
Amit Kumar et al. [38]	Ensembling method: three pretrained CNN models—DenseNet201, Resnet50V2, and Inceptionv3	COVID-19 positive and COVID-19 negative	COVID-19 +ve: 538 COVID-19 −ve: 468	95.7%
Gupta et al. [19]	InstaCovNet-19 (integrated stacking of InceptionV3, NASnet, Xception, MobileNetV2 and ResNet101)	COVID-19 and non-COVID	COVID-19: 361 Normal: 365	99.53%
Hussain et al. [20]	CoroDet	COVID-19 and normal	COVID-19: 2843 Normal: 1108	99.1%
Shelke et al. [15]	DenseNet-161	COVID-19 and pneumonia	COVID-19: 500 Pneumonia: 500	98.9%
Jain et al. [9]	ResNet-101	COVID-19 and viral pneumonia	COVID-19: 440 Viral pneumonia: 480	97.78%
**This study**	**DenseNet-121**	**COVID-19 and pneumonia**	**COVID-19: 1536** **Pneumonia: 5629**	**99.48%**

## Conclusion

Deep learning algorithm can aid healthcare workers in detecting COVID-19 with minimal processing of chest X-ray images. In this study, 2-class datasets were created which included COVID-19 and pneumonia images obtained from open sources. Several state-of-the-art pretrained neural networks that include ResNet50, DenseNet121, InceptionV3, VGG16, VGG19, Inception-ResNet-V2, and Xception were experimented using transfer learning technique. The best model turned out to be DenseNet-121 which accomplished an accuracy of 99.48%, followed by ResNet50 with a classification accuracy of 99.32%. This study summarizes that the detection models built using CNNs with transfer learning technique are able to perform good binary classification tasks on COVID-19 and pneumonia images. COVID-19 and viral pneumonia CXR images contain similar features which are challenging for the radiologist to interpret. However, the CNN model can easily learn the features in just a few epochs of training and classify the images correctly. The high accuracies obtained suggest that the deep learning models could find something distinctive in the CXR images and that makes the deep networks capable of distinguishing the images correctly. These trained models can effectively reduce the workload of medical practitioners and increase the accuracy and efficiency of COVID-19 diagnosis.

## Data Availability

All the data are available upon request from the corresponding author.

## Abbreviations

CNN: Convolutional neural network
COVID-19: Coronavirus disease of 2019
CT: Computed tomography
CXR: Chest X-ray
DeTraC: Decompose, Transfer, and Compose
DNA: Deoxyribonucleic acid
FFT: Fast Fourier transform
FN: False negatives
FP: False positives
GLCM: Gray-Level Co-Occurrence Matrix
GLDM: Gray Level Dependence Matrix
RNA: Ribonucleic acid
rRT-PCR: Real-time reverse transcription-polymerase chain reaction
SARS: Severe acute respiratory syndrome
SVM: Support vector machine
TN: True negatives
TP: True positive
VGG: Visual Geometry Group
